# Carotid body tumor: characteristics and surgical outcome

**DOI:** 10.1186/s13019-024-02951-0

**Published:** 2024-07-31

**Authors:** Fahmi Hussein Kakamad, Mihr Naif Mustafa, Shara Wahdaldeen Yasin, Shanga Sherzad Xalid, Ayoob A. Mohammed, Snur Othman, Dilan S. Hiwa, Hiwa O. Abdullah, Berun A. Abdalla, Hawkar A. Nasralla, Sasan M. Ahmed, Ayman M. Mustafa, Shko H. Hassan, Bushra O. Hussein

**Affiliations:** 1 College of Medicine, University of Sulaimani, Madam Mitterrand Street, Building 11, Apartment 50, Sulaymaniyah, Sulaymaniyah, Kurdistan 46001 Iraq; 2 Scientific Affairs Department, Smart Health Tower, Madam Mitterrand Street, Sulaymaniyah, Kurdistan Iraq; 3 Kscien Organization for Scientific Research, Hamdi Street, Azadi Mall, Sulaymaniyah, Kurdistan Iraq

**Keywords:** Carotid body tumor, Paraganglioma, Chemodectoma, Carotid bifurcation, Neuroendocrine tumor, Preoperative embolization

## Abstract

**Background:**

Carotid body tumors are uncommon neuroendocrine growths near the carotid bifurcation. While some advocate preoperative embolization to minimize bleeding, others avoid it due to complications. This study shares the experience of a single center in managing patients with carotid body tumors without practicing preoperative embolization.

**Methods:**

This was a cross-sectional study of patients with carotid body tumors managed between 2020 and 2024. Data were collected from the hospital’s registry. When necessary, routine blood tests, neck ultrasonography, and computed tomography scans were conducted. The tumors were categorized according to Shamblin’s classification. The average duration of follow-up was 20 months.

**Results:**

The study involved 25 patients, 22 (88%) females and 3 (12%) males. Their ages ranged from 27 to 85 years old. Twenty (80%) cases presented with neck swelling, and six (24%) had a positive medical history. Tumors were mainly on the right side (52%), with 20 (80%) showing ill-defined neck masses. Tumor sizes ranged from 1.5 to 7 cm, with Shamblin type II tumors being discovered in the majority of cases (72%). Types of tumors were significantly associated with the tumor size (p-value < 0.05). Blood transfusion was required in five cases (20%), three from type III and two from type II, with none from type I (p-value = 0.001). Temporary neurological deficits occurred in 3 cases (12%). No functional impairment or mortality was recorded.

**Conclusions:**

Carotid body tumors are rare tumors with an unknown etiology. Operation without practicing preoperative embolization may be feasible with an acceptable outcome.

## Background

Carotid body tumors (CBTs), also called paragangliomas or chemodectomas, are uncommon neuroendocrine growths emerging near the carotid bifurcation from glomus cells formed during embryonic neural crest development. They occur at a reported incidence of 1–2 cases per 100,000 people and represent approximately 0.6% of head and neck tumors. These tumors are typically benign, with malignant cases comprising less than 10% [1, 2]. The carotid body comes from the mesoderm of the third branchial arch, and the neural crest lineage is derived from the ectoderm. A typical carotid body is a pink, oval-shaped structure measuring around 6 × 4 × 2 mm. It is often believed, although possibly incorrectly, to be situated towards the back within the adventitia at the point where the common carotid artery bifurcates, and recent studies described periadventitial tissue as the exact location of the carotid body [3]. The gland receives its nerve supply from the glossopharyngeal nerve. It has the highest blood supply per gram of tissue compared to any other tumor, sourced from vasa vasorum, branches of the vertebral artery, and primarily from branches of the external carotid artery [4–6]. Histologically, the gland is made up of several lobules containing three different types of cells, each primarily responsive to hypoxia [7]. Type I cells, previously known as chief or glomus cells, secrete catecholamines and various immunoreactive peptides and are organized in nests called zellbalen. Under conditions of chronic hypoxia, the gland undergoes morphological changes, becoming hypertrophic and eventually hyperplastic. Type II cells, also referred to as sustentacular or sheath cells, surround the type I cells and exhibit characteristics of Schwann cells. Type III cells are sensory nerve endings originating from the sensory ganglion of the glossopharyngeal nerve and function as an afferent pathway for chemoreceptor reflexes that travel through the carotid sinus nerve to the respiratory control center. Recent findings have verified that all three cell types produce various neurochemicals that act as first and second messengers, influencing target cells through hormonal mechanisms [8–12]. Albrecht Von Haller first described the carotid body anatomically in 1743. Attempts to resect CBT were reported in the 1880s, but initial cases were unfortunate, with instances of intraprocedural hemorrhage or severe neurological complications leading to death. The first successful CBT resection in the United States was performed by Scudder in 1903. Despite these early challenges, even as late as 1957, mortality and morbidity rates were so high that Hayes Martin suggested in his textbook to stop resecting difficult (now known as Shamblin group III) tumors if the diagnosis was confirmed. Since then, advancements in imaging and vascular surgical techniques have significantly decreased mortality and morbidity rates [3]. By 1990, approximately 1000 cases had been documented in global literature [13].

While the exact cause of CBTs remains unclear, non-familial and familial factors have been identified. Non-familial factors include chronic hypoxic stimulation, as observed in individuals living at high altitudes or those with chronic heart or lung disorders. Familial cases, which constitute a significant proportion of CBTs, have been associated with genetic mutations, with several susceptibility genes identified through mapping studies [14].

There is considerable debate surrounding the etiology, the necessity of surgical intervention, and the outcomes linked with resecting CBTs. While some scholars advocate for preoperative embolization to minimize intraoperative bleeding, others forgo this step due to concerns about potential complications [14–16]. This study aims to share the experience of a single center in managing 25 cases of CBTs without practicing preoperative embolization. The eligibility of the references has been confirmed [17].

## Methods

### Study design and settings

A cross-sectional study was conducted on consecutive patients received during 45 months (from May 1, 2020, to February 1, 2024). Data were collected from the hospital’s registry. All patients underwent routine blood tests, neck ultrasonography, and a contrast-enhanced computed tomography (CT) scan if the diagnosis was uncertain. They were also examined before the operation for cranial nerve palsy. Patients were classified according to the Shamblin classification into types I, II, and III [5]. The average duration of follow-up was 20 months. The ethical committee of the College of Medicine at the University of Sulaimani approved the study, with registration number 149 dating back to January 21, 2024.

### Eligibility criteria

All patients diagnosed with CBTs who provided consent to undergo surgical intervention and also participate in this study were included. Patients with incomplete data, loss to follow up, or who did not provide consent to participate were excluded.

### Surgical intervention

Preoperative embolization was not performed in any cases due to the risk of cerebral vascular accidents, lack of enough expertise, and feasible intraoperative control of bleeding. Functional assessment was performed only for those with high blood pressure. The operations were performed under general anesthesia in the supine position, with the neck tilted to the contralateral side. A longitudinal incision (4 to 7 cm) was made in the anterior border of the sternocleidomastoid muscle. The common carotid artery proximal to the tumor was exposed and controlled. The dissection proceeded cranially with the direction of the external carotid artery (isolated first) to control the blood supply to the tumor. Risk of bleeding was minimized by putting a nonvascular clamp. Lastly, the internal carotid artery was isolated carefully to avoid injury to the hypoglossal nerve, which was recognized in all cases. Temporary neurological deficit was defined as any postoperative neurological deficit resolving within six months. A permanent neurological deficit was defined as a any postoperative neurological deficit lasting more than six months.

### Data analysis

The data were extracted from the database and transferred to an Excel sheet (Microsoft Excel version 2013). Mean, range, and frequency were calculated for all variables. Chi-square test was used for comparing the categorical data and the T-test for the numerical data. The significance level was set at less than 0.05.

## Results

**Fig. 1:**
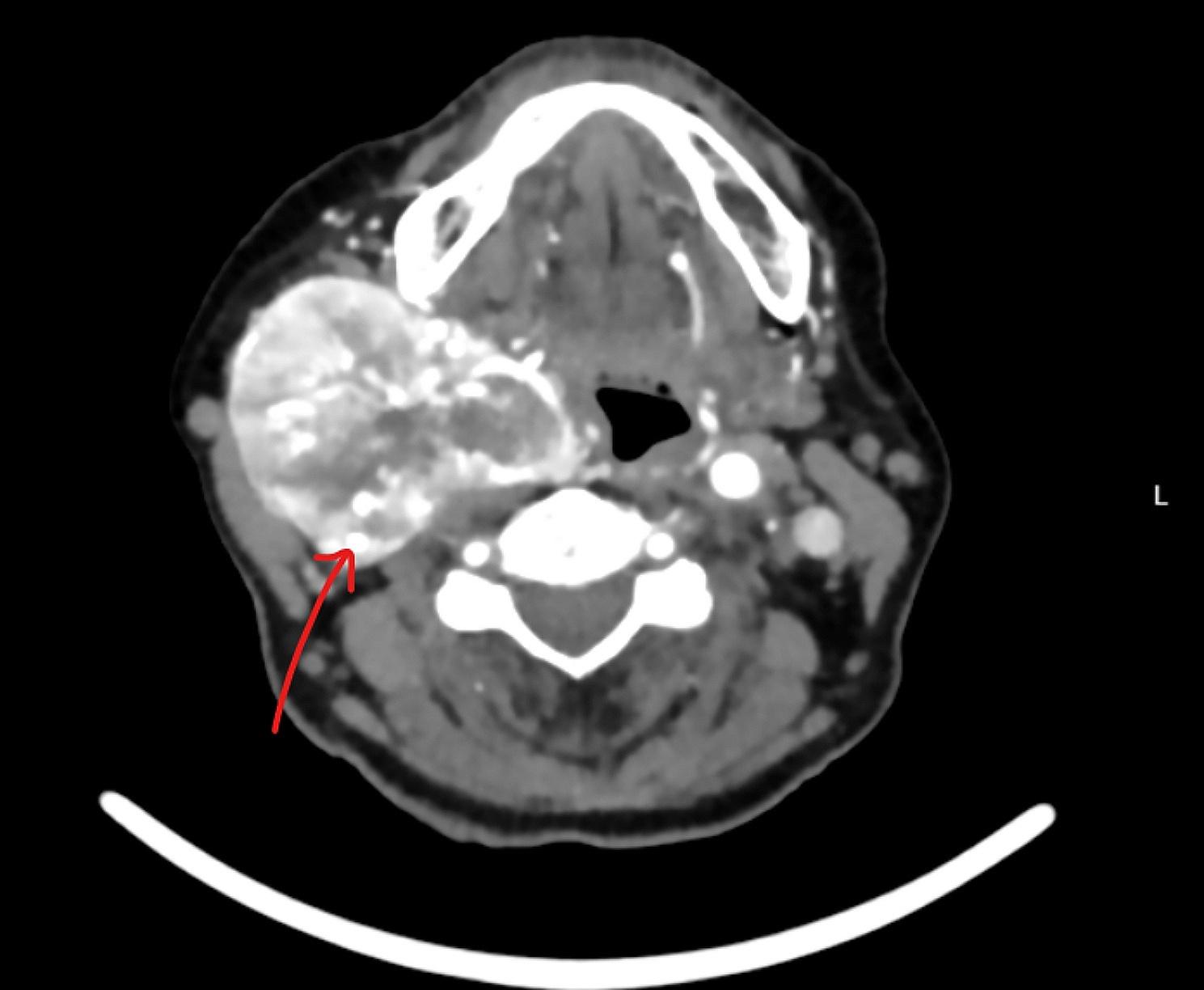
Computed tomography scan of the neck with intravenous contrast showing a large right side hypervascular mass splay of both internal and external carotid arteries with complete encasement of the internal carotid artery (the arrow)

The study comprised 25 patients, of whom 22 (88%) were female and the remaining three (12%) were male. Their ages ranged from 27 to 85 years, with a mean age of 52 years. All of them (100%) were from urban areas with low altitudes (less than 1000 m above sea level). Twenty cases (80%) presented with neck swelling, with pain noted in one case (4%); the disease was found incidentally in the remaining patients (5 cases, 20%). Past medical history was positive in 6 patients (24%), while one patient (4%) had a history of total thyroidectomy (Table 1). The tumor was found on the right side in 13 cases (52%) and on the left side in 11 patients (44%), while in one patient (4%), the disease was bilateral. One patient (4%) had a previous history of CBTs resection twice. In 20 cases (80%), the examination revealed an ill-defined neck mass with pulsation associated with tenderness in one case (4%). The examination was unremarkable in the remaining cases 5 (20%). Vital signs and routine laboratory investigations were normal, including complete blood count and erythrocyte sedimentation rate. Ultrasound examination was the only diagnostic test in two cases (8%); a CT scan was required for the provisional diagnosis in the remaining cases 23 ( 92%). The size of the tumors ranged from 1.5 to 7 cm, with an average size of 3.7 cm. The most common Shamblin type was type II 18 (72%) followed by type III 4 (16%) and type I 3 (12%) (Fig. 1). Types of tumors were significantly associated with the tumor size while not changing with the age of the patients (Table 2). Injury to the internal carotid artery was reported in 2 cases (8%); these were repaired directly using six zero-prolene sutures. Both cases were classified as type III. Lymph nodes were found and resected in 9 patients (36%); none were positive. Blood transfusion was required in five cases (20%) – three from type III and two from type II, with none from type I (p-value = 0.001). No permanent neurological deficits were reported, although temporary deficits were found in 3 cases (12%). The deficits were significantly associated with the type of tumor (p-value < 0.05) (Table 3). No functional impairment or mortality was recorded. No ICU admission was recorded.

**Table 1:** Past medical history association with carotid body tumor

Variables	*N* (%)
Congenital Heart disease	1 (4%)
Recurrent Thyroid tumor	1 (4%)
Crohn’s disease	1 (4%)
Multinodular goiter	1 (4%)
Thyroiditis	1 (4%)
Other disease	1 (4%)

**Table 2:** Types of the tumor in relation with the size and age of the patients

Shamblin classification	Average size (range) cm	Average age (range) year
Type I	1.8 (1.5-2)	60 (45–67)
Type II	3.4 (2–5)	49 (27–85)
Type III	6.4 (5–7)	63 (50–74)
	**P-value: 0.001**	**P-value: 0.051**

**Table 3:** Outcome of the operation in relation with the type of the tumor

Shamblin classification	Injury to hypoglossal nerve, number (%)	Injury to the marginal branch of the facial nerve number (%)	*P*-value
Type I	0 (0)	0 (0)	**0.001**
Type II	1 (5)	1 (5)	
Type III	0(0)	1(25)	

## Discussion

These tumors predominantly affect middle-aged females. In a study conducted by Sarookhani et al. involving 42 patients, 80.96% were female, with an average age of 54.41 years, which aligns closely with the findings of this study [18].

The condition typically manifests as a gradually enlarging, pain-free lump in the neck. It is often detected by chance during imaging procedures. Additional symptoms may involve tinnitus, persistent coughing, changes in voice quality, loss of sensation, Horner syndrome, prickling sensations, impairments related to cranial nerves, difficulty swallowing, localized sensitivity, increased heart rate, and sensitivity to light [18]. In this study, 80% (20 cases) presented with neck swelling, the remaining patients (5, 20%) were asymptomatic, and the disease was found accidentally. Other mentioned manifestations were not found; this may be due to a small sample size of this study.

CBT can be identified or suspected through a detailed history and physical examination, but confirming the diagnosis relies on imaging studies. These studies help determine the number of tumors, the condition of the extracranial and intracranial circulation, and the Shamblin classification of the tumor. Fine-needle aspiration CBT is challenging to interpret and potentially risky, so color duplex ultrasonography is a more suitable initial test for a lateral neck mass to rule out CBT [19, 20]. If this ultrasound supports or suggests the diagnosis, CT arteriography of the head and neck is the next logical step. This imaging can provide all necessary information for surgery if the tumor is solitary and there is no family history of such tumors. Patients with a personal or family history of neural crest tumors, or who have multiple paragangliomas identified through these studies, should undergo imaging of the mediastinum and retroperitoneum and assays to rule out accompanying pheochromocytomas. In some cases, computed tomography can be selectively useful. If further clarification of the Shamblin classification or intracranial circulation is needed, arteriography will likely resolve these issues [3]. In this study, the majority of the cases (92%) needed CT angiography of the neck for the diagnosis.

The CBTs were categorized for the first time in 1971 by Shamblin et al. into three groups based on their relationship with the adjacent blood vessels: small tumors that were confined to a specific area without significant attachment to the vessels (Type I), tumors that were attached to and partially encased the carotid vessels (Type II), and tumors that closely enveloped the carotid vessels (Type III) [5]. Type II is the most common type presented to vascular clinics [5]. This is in agreement with the findings of this study.

A retrospective analysis by Jiang et al. over a decade identified risk factors for postoperative cranial nerve injury in patients undergoing surgical treatment for CBTs. The study included 196 patients with 203 CBTs, revealing that 28.1% experienced cranial nerve injury after surgery. Significant correlations were found between postoperative nerve injury and factors such as Shamblin classification, external carotid artery ligation, internal carotid artery reconstruction, tumor volume, and blood loss. Multivariate logistic regression analysis identified Shamblin III classification and the number of lymph nodes removed as independent risk factors for nerve injury [21]. The present study showed no permanent neurological deficit, but a temporary neurological deficit was found in 12% of the cases. The lower rate of postoperative complications in this study might be explained by the lower rate of type III CBTs and the small sample size.

The malignancy rate of CBTs hovers around 5 to 7%, with a higher occurrence observed in younger patients, and sporadic tumors also exhibit this trend. Detection of CBT cells in regional lymph nodes raises suspicion of malignancy. This type of tumor can infiltrate nearby nerves, blood vessels, and even the skull. The external carotid artery is frequently affected. Metastases may spread to organs such as the brachial plexus, cerebellum, kidney, thyroid, breast, lungs, bones, and pancreas. Nerves, including the vagal, glossopharyngeal, accessory, and hypoglossal, which traverse the carotid sheath, may become involved. The clinical behavior of the tumor is the primary indicator of its malignancy. Although not definitively confirmed, several studies suggest a higher occurrence of malignant CBT in females [18, 22]. In this study, no malignant tumor was reported.

Surgical removal is the primary treatment option, as the tumor carries malignant potential and should be excised before it grows larger and becomes more challenging to remove. As the tumor exhibits high vascularity, intraoperative bleeding emerges as the primary concern, and the tumor’s characteristics hinder the efficacy of electrosurgery. While the tumor does not respond well to radiotherapy, it may be considered to control its growth in cases where surgery is not feasible [5]. Beyond medical considerations, certain risks make surgery unacceptable. These include: (1) a patient with a cranial nerve or sympathetic trunk injury on one side may not be a suitable candidate for the removal of a CBT on the opposite side; (2) removing a CBT will unavoidably denervate the carotid sinus on that side. If the contralateral CBT is also removed, it can lead to refractory hypertension due to the complete loss of baroreception [23]. All of the cases in this study were managed by surgical resection under general anesthesia. Risks of bleeding were minimized by applying several non-vascular clamps, and frequently, the angle of dissection was changed.

Surgical complications, both immediate and delayed, encompass early stroke, necessity for emergency hematoma drainage, temporary impairment of cranial nerves, and delayed issues such as Horner’s syndrome, pseudoaneurysm, permanent cranial nerve damage, and delayed stroke [18, 24].

Mortality rates from CBT range from zero to 5.7%. Deaths typically result from local extension before surgery, intraoperative stroke, and blood loss. This study didn’t document any cases of mortality [5, 25].

## Conclusion

Carotid body tumors (CBTs) are rare tumors with an unknown etiology. Operation without practicing preoperative embolization may be a feasible management with an acceptable rate of morbidity and mortality.

## Data Availability

The data that support the findings are available from the corresponding author, upon request.
